# Phytosterol Profiles, Genomes and Enzymes – An Overview

**DOI:** 10.3389/fpls.2021.665206

**Published:** 2021-05-19

**Authors:** Sylvain Darnet, Aurélien Blary, Quentin Chevalier, Hubert Schaller

**Affiliations:** Plant Isoprenoid Biology Team, Institut de Biologie Moléculaire des Plantes du CNRS, Université de Strasbourg, Strasbourg, France

**Keywords:** phytosterol, cholesterol, algae, plant, eukaryote, prokaryote

## Abstract

The remarkable diversity of sterol biosynthetic capacities described in living organisms is enriched at a fast pace by a growing number of sequenced genomes. Whereas analytical chemistry has produced a wealth of sterol profiles of species in diverse taxonomic groups including seed and non-seed plants, algae, phytoplanktonic species and other unicellular eukaryotes, functional assays and validation of candidate genes unveils new enzymes and new pathways besides canonical biosynthetic schemes. An overview of the current landscape of sterol pathways in the tree of life is tentatively assembled in a series of sterolotypes that encompass major groups and provides also peculiar features of sterol profiles in bacteria, fungi, plants, and algae.

## Introduction

Sterols are mandatory components of eukaryotic life as building blocks of cellular membranes and as bioactive signals both functions having specific molecular structural requirements (Schaller, 2010; Darnet and Schaller, 2019). The sterol repertoire, which is built from the C_30_H_50_O triterpenic committed precursors cycloartenol and lanosterol is part of the ubiquitous terpenome that is inseparable from the origin of life (Ourisson and Nakatani, 1994), just as DNA, RNA, and proteins. Sterols are also found in some bacterial groups but not in archaea (Salvador-Castell et al., 2019). Hopanoids in bacteria are considered as functional triterpenic counterparts of sterols in eukaryotes, but their presence is not strictly restricted to prokaryotes since some lycophyte species synthesize hopanoids (Kushiro and Ebizuka, 2010). Such an overlapping distribution of structurally different triterpenes and terpenogenic enzymes at play in distantly related taxa illustrates the fact that those pathways are quite ancient acquisitions, which have evolved through multiple mechanisms (Keeling and Palmer, 2008). The diversity and complexity of sterol pathways and the evolution of enzymes is closely associated to the rise of oxygen on earth about two billion years ago (Holland, 2002; Summons et al., 2006). In fact, the enzymatic conversion of squalene to diploptene (the simplest hopanoid) in bacteria is a one-step dioxygen-independent biosynthetic process, whereas the nineteen-enzymatic step conversion of squalene into 24-ethylsterols in viridiplantae requires 12 molecules of dioxygen (Nes, 2011; Gold et al., 2017). The analysis and dating of hydrocarbon biomarkers particularly steranes, which are molecular fossils produced by the diagenetic transformation of sterols, have helped to apprehend the importance of certain eukaryotic groups in geological periods. For instance, isopropylcholestane is debated as the most probable marker of chlorophyta in neoproterozoic sediments (Bobrovskiy et al., 2021).

The elucidation of sterol pathways from biosynthetic to genetic studies has attracted immense research efforts over the last decades (Cornforth, 2002). Major findings from the initial cyclization of the oxidized squalene (3*S*)-2,3-oxidosqualene into steroidal triterpenols (cycloartenol, lanosterol) to their conversion into cholesterol, ergosterol and phytosterols and the biological significance of those have been assembled in a global picture proposed for mammal, invertebrate, fungi, and plant model species (Nes, 2011; Brown et al., 2018; Moreau et al., 2018). The understanding of accurate biosynthetic processes for example with chemical radiotracers (Cornforth, 2002) preceded the characterization of corresponding enzyme activities but was limited to a small number of laboratory-compatible organisms. The growing access to genome mining is now rapidly broadening the understanding of the origin and distribution of sterol biosynthetic capabilities in the tree of life. The best example when considering sterol pathways is the widespread occurrence of cycloartenol (and cycloartenol synthase, CAS) in prokaryotes (Bode et al., 2003) and eukaryotes like *Dictyostelium discoidum* (Godzina et al., 2000; Meyer et al., 2002) or Naegleria (Raederstorff and Rohmer, 1987). In fact, novel and massive genome and metagenome analysis coupled with *ad hoc* functional validation assays will facilitate the access to yet undisclosed sterol biosynthetic enzymes or more generally new pathways and therefore new aspects of cellular homeostasis.

Here, the purpose is to provide an overview of main aspects of sterol profiles and corresponding enzymes from various organisms including plants and algae but not exclusively, and to tentatively classify organisms into sterolotypes, considering a wealth of genetic, biochemical, and phylogenomic data.

## Canonical Features of Sterol Biosynthesis in Eukaryotes

**FIGURE 1:**
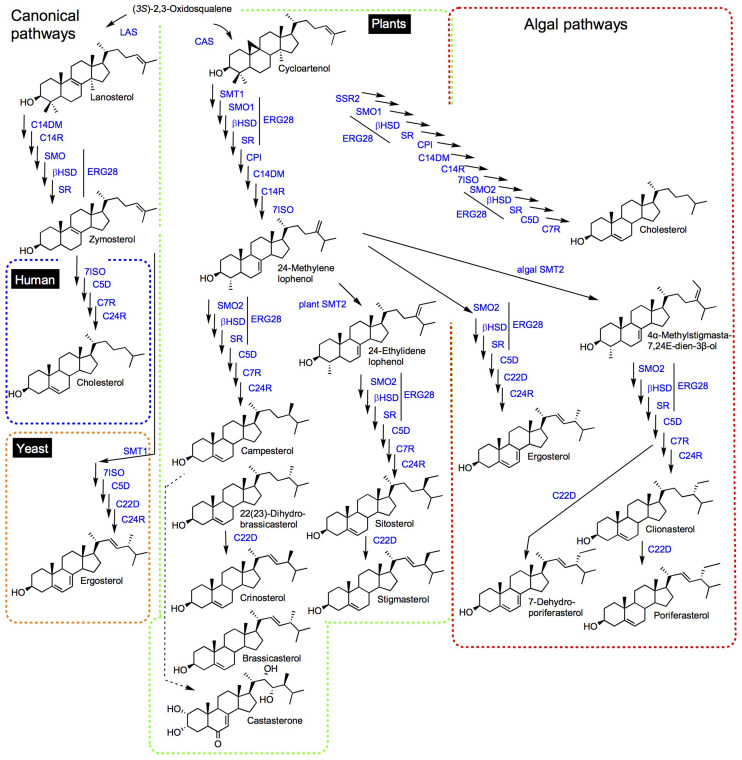
Synthetic view of sterol pathways. Dashed lines discriminate mammalian (blue), fungal (red), and plants (green) canonical pathways. The so-called non-canonical pathways shown for algae is framed in mixed red and green colors to point out Δ^5,7^-sterols in both fungal and algal groups, and to recall common side chain modifying enzymes SSR2 and SMTs. Substrate-product enzymatic conversion is visualized with a single arrow. The dashed line between campesterol and castasterone (brassinosteroids) represents multiple steps. Sterol names are non-systematic names. The comprehensive IUPAC recommendations and conventional system for sterol nomenclature based on cholestane, ergostane, and stigmastane scaffolds, and the associated stereochemistry is given in Moss (1989) and also commented in Nes (2011). LAS, lanosterol synthase; CAS, cycloartenol synthase; C14DM, sterol-14-demethylase; C14R, sterol-14-reductase; SMO, sterol methyl oxidase; βHSD, 3β-hydroxysteroid dehydrogenase/C4-decarboxylase; SR, sterone ketoreductase; 7ISO, sterol-7(8)-isomerase; C5D, sterol-5(6)-desaturase; C7R, sterol-7-reductase; C24R, sterol-24-reductase; SMT1, sterol-C24-methyltransferase; SMT2, sterol-C28-methyltransferase; CPI, cyclopropyl isomerase; and C22D, sterol-22-desaturase.

**FIGURE 2:**
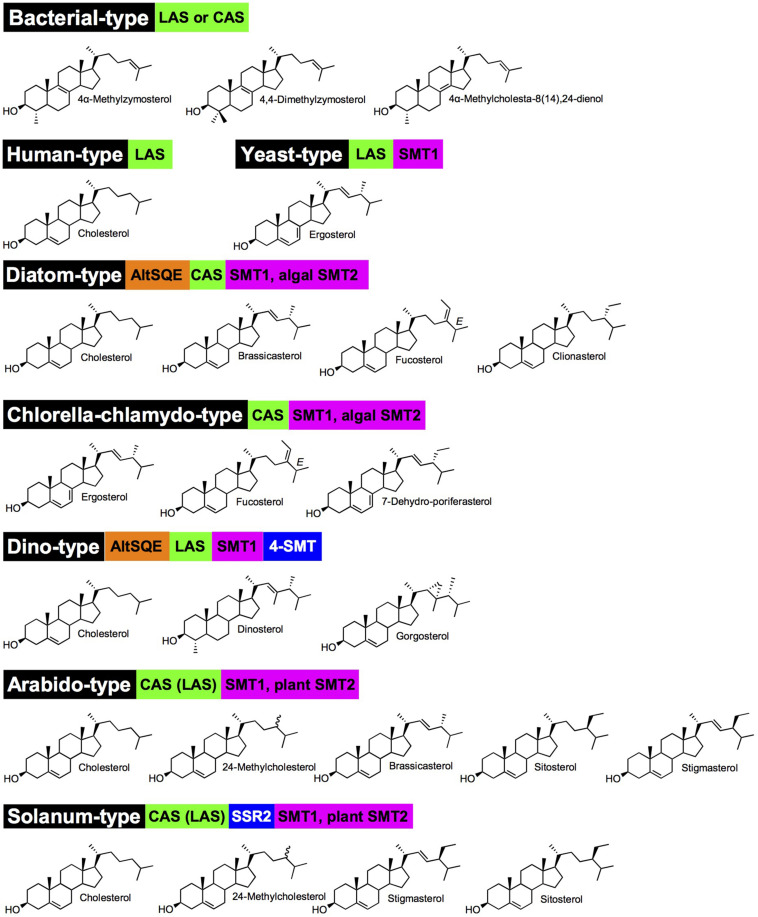
Major sterolotypes defined in living organisms. Names in black boxes are chosen according to species or taxonomic groups for which a sterol pathway is well documented. Green boxes indicated whether a CAS (cycloartenol synthase) or LAS (lanosterol synthase) defines a sterol pathway; brackets are for minor contribution of a pathway as currently understood. Orange boxes are marking an AltSQE, alternative squalene epoxidase (canonical SQE otherwise not indicated in sterolotypes). Blue and magenta boxes are for sterol side chain modifications by SSR2 (sterol side chain reductase 2), SMT, sterol-C-methyltransferases. Major or representative sterols are represented for each sterolotype. Nomenclature as in Figure 1. Arabido-type and Solanum-type exhibit the epimeric pair of campesterol (C24α-methylcholesterol) and 22(23)-dihydrobrassicasterol (C24β-methylcholesterol) represented by 24-methylcholesterol.

Enzymes implied in the post-squalene pathways to sterols in model organisms (mammals: human, mouse; fungi: *Saccharomyces cerevisiae*; and chlorophyta: *Arabidopsis thaliana*) have been quite comprehensively described and orthologous or paralogous enzymes were found in related species or taxonomic groups based on functional genomics (Bloch, 1989; Benveniste, 2004; Schaller, 2010; Nes, 2011; Sonawane et al., 2016; Darnet and Schaller, 2019). Initial biosynthetic studies have led to the characterization of enzyme activities responsible for the successive substrate-product bioconversions identified in incorporation experiments or by the targeted action of chemical inhibitors (Guo et al., 1997). Organic analytical chemistry also preceded functional genomics to unveil a major pathway for the production in C_5_ isoprenic units (isopentenyl diphosphate, dimethylallyl diphosphate) in prokaryotes and some eukaryotes, condensed in linear terpenic precursors (Rohmer, 1999). The occurrence of this so-called methylerythritol 4-phosphate (MEP) pathway in prokaryotes and plastids of the chlorophyta and apicomplexa, along with the classical (first described) mevalonate pathway in vertebrates, invertebrates, fungi and plants is now mapped on the tree of life with an increasing precision and consequently with exceptions or alternatives (Lombard and Moreira, 2011). Forward and reverse genetic screens and genome mining provided scientists with a compendium of genes implied in the conversion of squalene into sterol end-products like cholesterol, ergosterol, stigmasterol, and poriferasterol in human, yeast, land plants and algae, respectively (Figure 1). In brief, human (or mammals and other groups of sterol autotroph animals) and fungi use lanosterol as a committed precursor to produce cholesterol, a C_27_ product with an 8-carbon atom side chain, and ergosterol, a C_28_ product with one additional exocyclic carbon atom at C-24 added by a sterol-C-methyltransferase [SMT, here a zymosterol-C24-methyltransferase (SMT1)], respectively. Differences between mammals and fungi includes also a sterol-C22(23)-desaturase (C22D) present in yeast only and a sterol-7-reductase (C7R) absent from yeast only, indicative of gene gain and loss as a powerful driver of sterol profile diversification. The *A. thaliana* sterol pathway as a model for higher plants displays very strong differences with human and yeast canonical pathways. Plants use cycloartenol as a committed precursor. A further sterol diversification by the action of two distinct SMT1 and SMT2 is responsible for the formation of 24-methylsterols and 24-ethylsterols (C_29_ products), respectively, (Bouvier-Nave et al., 1998; Schaeffer et al., 2001; Nakamoto et al., 2015). The Arabidopsis pathway also includes a C22D of the same cytochrome P450-dependent monooxygenases group (CYP710A) as the fungi enzyme (Morikawa et al., 2006). Arabidopsis has also evolved two distinct sterol methyl oxidases SMO1 and SMO2 acting non-consecutively during the course of the conversion of cycloartenol into the complex 24-alkyl-Δ^5^-sterol mixture typical of plants (Darnet and Rahier, 2004). Conversely, a single SMO in fungi and mammals contributes to the C4-demethylation complex of enzymes [including in addition to SMO, a 3β-hydroxysteroid dehydrogenase/C4-decarboxylase (βHSD), a sterone ketoreductase (SR), and a tethering ERG28 protein] that removes successively both methyl groups at C-4 (Figure 1; Bouvier et al., 2005). In addition, the mandatory biosynthesis of phytosterols from cycloartenol (Babiychuk et al., 2008) requires a cyclopropyl sterol isomerase (CPI; Men et al., 2008), which is also present in some protists like *D. discoidum* that use the cycloartenol pathway (Lovato et al., 2000). Mammalian, fungi and plant sterol pathways therefore define major sterolotypes based on sterol profiles and the associated enzymes like lanosterol synthase (LAS) in mammals, LAS and SMT1 in fungi, and CAS and SMT1 and SMT2 in higher plants (Figure 2). Gene gain or loss differentiate these pathways, otherwise sharing significant orthology and paralogy thus enabling functional cloning in yeast of human and plant enzymes for biochemical complementation or metabolic interference (Corey et al., 1993; Lecain et al., 1996; Husselstein et al., 1999). Those canonical pathways have been quite extensively reviewed (Benveniste, 2004; Schaller, 2010; Nes, 2011; Brown et al., 2018; Moreau et al., 2018). Very strikingly, further gene discovery in sterol metabolism still relies on direct and possibly challenging biochemical approaches. The recent finding of aspartyl ergosterol and the set of enzymes promoting conjugation and deconjugation of ergosterol with amino acids represent a prominent example in the field. This novel group of sterols restricted to fungi (except in *S. cerevisiae* where the gene is absent) is formed by the action of non-canonical tRNA acyltransferases (Yakobov et al., 2020).

**FIGURE 3:**
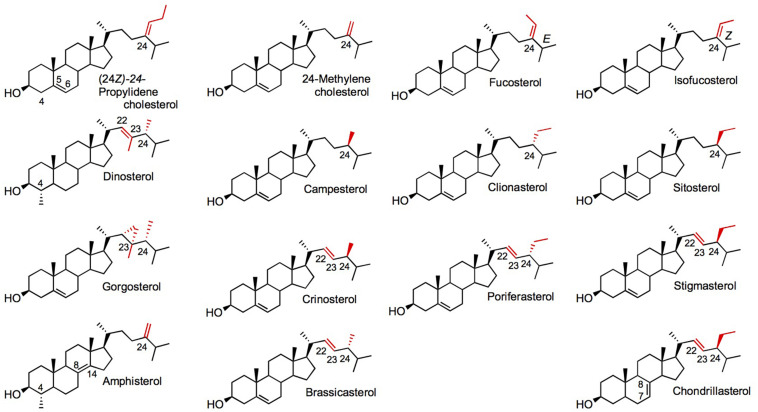
Diverse sterols found in algae and plants. Side chain C22, C23, and C24 positions are in red. Chiral substituents at C24 of the sterol side chain are C24α for a methyl or ethyl group in front: campesterol, sitosterol, crinosterol, stigmasterol, chondrillasterol; or C24β for a methyl or ethyl group in back: dinosterol, clionasterol, gorgosterol, poriferasterol. Nomenclature as in Figure 1.

**FIGURE 4:**
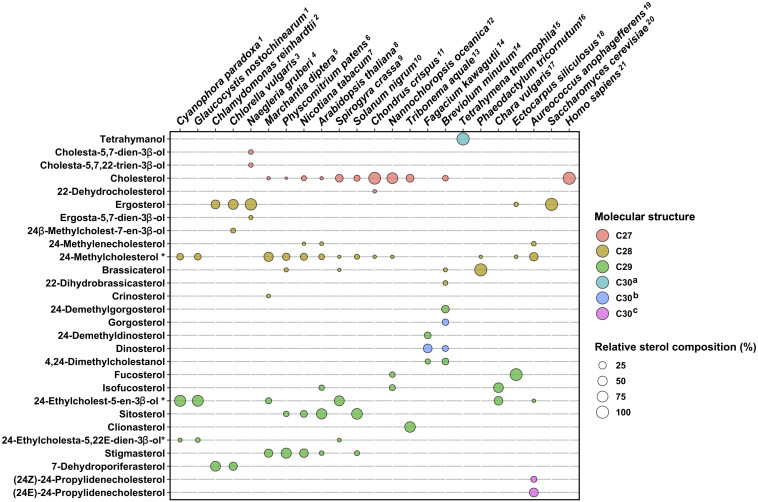
Sterol profiles in algae and plants. Sterol compositions of twenty species are plotted on a schematic diagram, which indicates proportions in percent and molecular structures by color codes. C_27_, sterols bearing a C_8_-side chain (e.g., cholesterol); C_28_, C_9_-side chain (e.g., ergosterol); and C_29_, C_10_-side chain (e.g., fucosterol). C_30_^*a*^, C_30_H_50_O direct cyclization products of (3*S*)-2,3-oxidosqualene (e.g., tetrahymanol). C_30_^*b*^, sterol products with additional methyl groups on the tetracyclic moiety and on the side chain (e.g., dinosterol). C_30_^*c*^, sterol products with a C_11_-side chain (e.g., propylidene sterols). Sterol compositions as percent of the total were found in published references indicated by upper case numbering of species. Sterols contributing less than 2% of a total were not considered for clarity of this schematic representation. 1, Leblond et al. (2011); 2, Miller et al. (2012); 3, Akihisa et al. (1992); 4, Raederstorff and Rohmer (1987); 5, Chiu et al. (1985); 6, Morikawa et al. (2009); 7, Schaller et al. (1998); 8, Schaeffer et al. (2000); 9, Mitova et al. (1999); 10, Bhatt and Bhatt (1984); 11, Tasende (2000); 12, Lu et al. (2014); 13, Mercer et al. (1974); 14, Lu et al. (2020); 15, Conner et al. (1982); 16, Rampen et al. (2010); 17, Patterson and Nes (1991); 18, Mikami et al. (2018); 19, Giner et al. (2009); 20, Lamacka and Sajbidor (1997); and 21, Chakrabarti et al. (2017). *Tetrahymena thermophila*, *Saccharomyces cerevisiae*, and *Homo sapiens* are shown as single pathway end-product species. Sterols tagged with an asterisk * are C-24 epimeric mixtures.

Besides canonical pathways, sterol biosynthetic processes in non-model eukaryotes are often overlooked, since sterol profiles only are reported but genomic data are just emerging. This is particularly the case for the vast group of algae (chromalveolate microalgae, rhodophyta, and green algae, etc.), which had been described for their sterol content in authoritative reports years ago (Patterson, 1971, 1974) or more recently (Kumari et al., 2013; Volkman, 2016; Figure 1). Major catalysts that generate the above-mentioned sterol diversity are SMT and sterol-Δ^24^-reductases (C24R) or C22D implied in the modification of the sterol side chain (Nes, 2011). These enzymes are of pivotal importance for producing the epimeric 24-alkyl-sterols in different groups. The big picture obtained from decades of analytical work shows a taxonomy that recapitulates sterol stereochemistry at C24, with algae producing C24β-ethylsterols (clionasterol, poriferasterol) and higher plants producing C24α-ethylsterols (β-sitosterol, stigmasterol; Goad and Goodwin, 1972; Figure 1). Epimeric pairs of sterols like campesterol (C24α) and 22(23)-dihydrobrassicasterol (C24β), sitosterol (C24α), and clionasterol (C24β), stigmasterol (C24α), and poriferasterol (C24β; Figures 1, 3) can be distinguished by NMR spectroscopic methods (Rubinstein et al., 1976). Studies with biological membrane mimics have shown equivalent structural behavior of 24-ethyl epimers (Marsan et al., 1996). The unequal distribution in taxa of those epimers is raising the interesting question of stereochemistry possibly recapitulating biology, as suggested from the progress in brassinosteroid signaling and biosynthesis made at a fast pace since 1996 (Li et al., 1996). In Arabidopsis, campesterol (24α-methylcholesterol) is the committed precursor of brassinosteroids through a C22 hydroxylation pathway whereas the epimeric 22(23)-dihydrobrassicasterol has not been reported as an active brassinosteroid precursor (Fujita et al., 2006; Bajguz et al., 2020). The astonishing diversity of sterol side chain biosynthetic modifications will be further scrutinized thanks to the growing number of eukaryotic genome and metagenome sequencing initiatives. The current global picture of sterol profiles across phyla shows a widespread distribution of C_27_, C_28_, and C_29_ sterols whereas C_30_ products are restricted to a few species (Figure 4).

## Squalene Metabolism Into Committed Triterpenes

**FIGURE 5:**
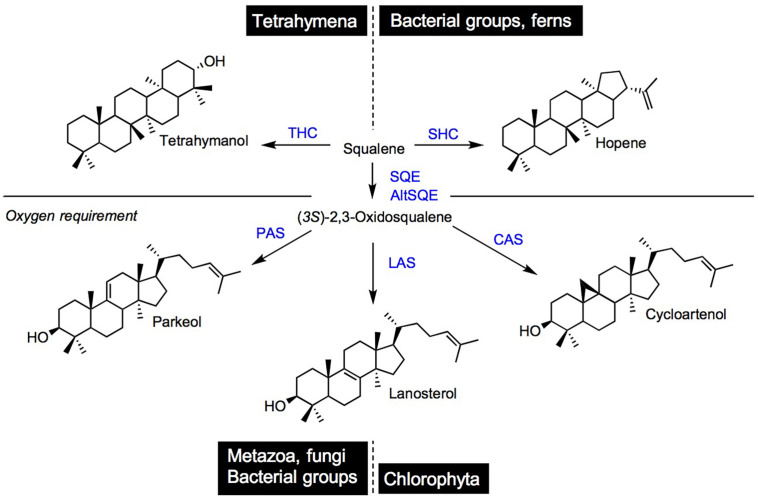
Cyclization products of squalene and (3*S*)-2,3-oxidosqualene in species and taxonomic groups. THC, tetrahymanol synthase; SHC, squalene hopene cyclase; SQE, squalene epoxidase; AltSQE, alternative squalene epoxidase; PAS, parkeol synthase; LAS, lanosterol cyclase; and CAS, cycloartenol cyclase.

Squalene is the committed C_30_ isoprenic precursor of tetrahymanol and hopanoids, and after its conversion into (3*S*)-2,3-oxidosqualene, of steroidal (cycloartenol, lanosterol, and parkeol) and non-steroidal triterpenoids (Figure 5). While squalene- and (3*S*)-2,3-oxidosqualene-derived non-steroidal triterpenoids exhibit a skeletal chemical structure distinct from that of steroidal compounds (Xu et al., 2004), tetrahymanol and hopanoids do exert membrane structural functions classically assigned to sterols, as described below. Tetrahymanol is produced in the ciliate *Tetrahymena pyriformis* by a squalene-tetrahymanol cyclase (THC; Saar et al., 1991; Wiersma et al., 2020). A *S. cerevisiae* loss-of-function mutant impaired in ergosterol biosynthesis was able to grow autotrophically when expressing the *Tetrahymena* THC, indicating therefore a functionality of tetrahymanol as a sterol surrogate (Wiersma et al., 2020). Some bacteria are also tetrahymanol producers, however, their biosynthetic process leading to tetrahymanol requires first a squalene-hopene cyclase (SHC) that generates a product on which the bacterial tetrahymanol synthase (THS) performs additional ring expansion of the triterpenic product (Banta et al., 2015). Consequently, THC and THS enzymes define distinct systematic groups, which possess identical triterpene products resulting from evolutionary convergent processes.

Hopanoids are widely distributed in ecosystems. This is shown by their presence in sediments as stable hopane derivatives (or geohopanoids), which are markers of life of particular interest in petroleum geochemistry (Ourisson et al., 1982). These hopanoids are found in many prokaryotic groups (bacteria, cyanobacteria) and are quite often designated as “sterol surrogates” acting as phospholipidic bilayer stabilizers or reinforcers (Bird et al., 1971; De Rosa et al., 1971; Rohmer et al., 1979; Delgado-Baquerizo et al., 2018). This was established in a series of *in vitro* experiments showing that diplopterol and cholesterol achieved identical effects on phase behavior and ordering of sphingolipids in model membranes (Saenz et al., 2012; Mangiarotti et al., 2019). Hopanoids have attracted considerable research focus on plant-bacteria interactions particularly in legume-rhizobia symbiotic root nodulation since the initial characterization of hopanoid-lipid A conjugates (Silipo et al., 2014; Belin et al., 2018). Hopanoid producers are found within eukaryotes, in lichens and ferns producing both squalene-derived products, and (3*S*)-2,3-oxidosqualene-derived sterols just like canonical land plants. A cycloartenol to β-sitosterol pathway was described in *Selaginella* (Weng and Noel, 2013) and in *Azolla filiculoides* (Brouwer et al., 2015). Genome mining in these species provided new observations on fern-cyanobacteria symbiosis, with the identification of the cyanobacterial origin (from *Nostoc azollae* also called *Anabaena azollae*) of the lycophytic SHC (Pereira and Vasconcelos, 2014; Li et al., 2018). Functions of hopanoids as membrane lipids have been more specifically assigned in oxygenic photosynthetic cyanobacteria to the efficient protection through compartmentation, of the essential nitrogenase implied in nitrogen fixation, which is a dioxygen-sensitive process. At the most global scale of the oceans it is proposed that hopanoids are recruited to optimize the efficiency of cyanobacterial diazotrophs (Cornejo-Castillo and Zehr, 2019). This is of course reminiscent of the role of hopanoids as reinforcers of the vesicular structure that protects the site of nitrogenase activity in plant-rhizobia symbiotic relationships (Berry et al., 1993).

The mandatory biosynthetic route to sterols through cycloartenol and not lanosterol in plants was established in a series of comprehensive biosynthetic and genetic studies (Corey et al., 1993; Benveniste, 2004; Babiychuk et al., 2008; Gas-Pascual et al., 2015). However, extensive genome mining based on protein motifs and specific amino acid residue conservation or variation unveiled the presence of LASs in many plant species (Kolesnikova et al., 2006; Suzuki et al., 2006). The significance of a catalytically functional LAS from plants, when expressed in a heterologous yeast, is yet asking for more studies since a loss of function *las* allele from Arabidopsis displayed no morphogenetic inhibitions (Ohyama et al., 2009). In *Euphorbiaceae*, a LAS ortholog is producing loads of lanosterol in the cytoplasm of laticifers, in combination with other triterpene synthases like CAS and a butyrospermol synthase (Forestier et al., 2019). *Solanaceae* do have a LAS, but produce high amounts of cholesterol through the cycloartenol route like it is the case for other phytosterols. In fact, the lanosterol-unrelated cholesterol is a precursor of solanine and chaconine and of some other steroidal glycoalkaloids in tomato and potato, accounting for some 15–20% of total sterols. Besides a canonical plant sterol-Δ^24(28)^-reductase (Figure 1), a solanaceae-specific sterol-Δ^24(25)^-reductase also named sterol side chain reductase 2 (SSR2) reduces the Δ^24(25)^ of the 8-carbon side chain of cycloartenol and desmosterol, yielding cholesterol (Sawai et al., 2014; Sonawane et al., 2016). Consequently, a sterol pathway based on a CAS, SSR2, and SMT1 and SMT2 defines a major sterolotype among eukaryotes (Figure 2). The solanaceae *Withania somnifera* (Indian ginseng or ashwagandha) has a sterol-Δ^24^-reductase coined 24ISO performing the conversion of 24-methylenecholesterol to 24-methyldesmosterol and consequently channeling the latter substrate into the specific biosynthetic segment for withanolides (Knoch et al., 2018).

## Minimal Sterol Pathway in Prokaryotes

Sterols are quite often classified as eukaryotic lipids but in fact some bacterial species synthesize sterols. A prominent example of a bacterial pathway is given by the methylotroph *Methylococcus capsulatus*, which produces unique Δ^8(14)^ -sterols (Bouvier et al., 1976). *M. capsulatus* genes encoding a squalene epoxidase, a LAS and a lanosterol-14-demethylase have been identified (Lamb et al., 2007). This bacterial pathway converts lanosterol into a series of metabolites of which 4α-methylcholesta-8(14),24-dienol could be the end-product, which implies a set of enzymes for the demethylation at C-4, reduction at C-24, and isomerization of the lanosterol double bond 8(9) into 8(14). The discovery of C-4 demethylation proteins SdmA (a Rieske-type oxygenase) and SdmB [a NAD(P)-dependent reductase], which convert lanosterol into the corresponding 4-demethylated product shows that a sterol C4-demethylation process has evolved independently in prokaryotes and in eukaryotes (in which a C4-methyl group oxidation is carried out by enzymes from the fatty hydroxylase group; Lee et al., 2018). Other bacteria have evolved other minimal sterol pathways. *Gemmata obscuriglobus* is a planctomycetes that contains a (3*S*)-2,3-oxidosqualene cyclase (OSC) responsible for the formation of the isomers lanosterol and parkeol, without further metabolization of these steroidal triterpenes (Pearson et al., 2007). *Stigmatella aurantiaca* is a myxobacterium that produce sterols from cycloartenol (Bode et al., 2003). Genomic and metagenomic surveys pointed out the presence of OSCs in several bacterial phyla (bacteroidetes, planctomycetes, proteobacteria, and some others), broadening thus the evolutionary success of a bacterial type of sterol pathway (Wei et al., 2016). Although scarce some recent data indicate possible symbiotic interactions at the sterol metabolism interface: the combination of genomics and isotopic ratio mass spectrometry of sterols has been exploited in a probably unique study suggesting a metabolic cooperation between a methanotroph endosymbiont and its eukaryotic host, the mussel *Bathymodiolus genus*. As a matter of fact, the isotopic ratio δ^13^C measured for the host cholesterol suggest a direct biogenetic link with methane from deep sea environments. In addition, transcripts for steroidogenic enzymes from the endosymbiont -which would provide the upstream biosynthetic pathway- and the host -which would provide the downstream biosynthetic pathway- were colocalized at the gills of the bivalves in *in situ* hybridization experiments (Takishita et al., 2017).

Cyanobacteria and other oxygenic photosynthetic bacteria from the genera *Anabaena*, *Nodularia*, *Nostoc*, *Spirulina*, *Chlorogloeopsis*, *Phormidium* conceal some sterol producing species as it was debated in the sixties especially with the detection of 24-ethylcholesterol (De Souza and Nes, 1968) and reviewed more recently (Volkman, 2003, 2016). Biosynthetic studies showing the conversion of isoprenic precursors to sterol products were not recently reported in any of the common cyanobacteria. *Phormidium autumnale* was shown to produce sterols albeit in heterotrophic culture systems compatible with an industrial scale-up (Fagundes et al., 2018). Metagenomic analysis of prokaryotic sequences pointed out OSC genes in some other genera of cyanobacteria like *Planktothrix* or *Prochloron* (Wei et al., 2016), but not in the genome of one of the most popular cyanobacterium *Synochocystis* sp. (Kaneko et al., 1996).

## Sterol Auxotrophy in Ciliates

**FIGURE 6:**
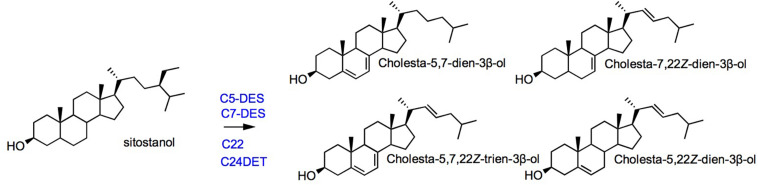
Sterol metabolization reactions in the ciliate *Tetrahymena*. The conversion of sitostanol is shown [adapted from Najle et al. (2013)]. C5-DES, sterol-5(6)-desaturase; C7-DES, sterol-7-desaturase; C22-DES, sterol-22-desaturase; and C24DET, sterol-24-de-ethylase enzyme abbreviations from Najle et al. (2013).

*Tetrahymena* spp. and *Paramecium tetraurelia* are sterol auxotrophs that develop on dietary sterols unless degraded environmental conditions like starvation switches on a capacity to make tetrahymanol (Raederstorff and Rohmer, 1988; Najle et al., 2020). The enzymes recruited for metabolization of exogenous sterols are a canonical sterol-C5-desaturase (Poklepovich et al., 2012) belonging to the fatty acid hydroxylase group (Sperling et al., 2003), a C24-dealkylase (or sterol-24-de ethylase) acting on the sterol side chain (Tomazic et al., 2011, 2014), and a Rieske Oxygenase performing a sterol-C7-desaturation (Nusblat et al., 2005; Najle et al., 2013). Enzymes of the same type are found in distant sterol-auxotroph, namely, insects, and nematodes (Zhou et al., 2020). This observation adds on typical cases of convergent evolution, here for a functional remodeling of phytosterols, by enzymes most probably acquired by horizontal gene transfer (Darnet et al., 2020). Genome wide investigations in *Tetrahymena* led to the identification of a sterol-22-desaturase belonging to the super family of fatty acid hydroxylases, and not to the canonical P450 monooxygenases (Najle et al., 2020). The multiple desaturations of exogenous cholesterol by *Tetrahymena thermophila* (Figure 6) is reminiscent of the conversion of dietary cholesterol into dafachronic acids – a class of signaling steroids- by *Caenorhabditis elegans*, a distantly related sterol-auxotroph (Zhou et al., 2020). Enzyme evolution and innovations (Newton et al., 2018) particularly in the super group of fatty acid hydroxylases is not restricted to ciliates when it comes to the post-squalene pathway. An enzyme of the fatty acid hydroxylase type was shown to use squalene as a substrate to produce (3*S*)-2,3-oxidosqualene in a diatom, therefore named alternative squalene epoxidase (AltSQE; Pollier et al., 2019).

## Lanosterol to 4-Methylsterol Pathway in Dinoflagellates

Dinoflagellates from the SAR group (comprising stramenopila, alveolate, and rhizaria) shown in the new tree of eukaryotes (Burki et al., 2020) exhibit very striking sterol biosynthetic peculiarities. This group is widespread in aquatic ecosystems and is most importantly hosting symbiotic species required for the remarkable biology of cnidaria and especially reef-building corals, which depend on their symbionts for nutrients and sterols (Lu et al., 2020). The sterol supply to an anemone from its *symbiodinaceae* symbionts was shown to be associated with a symbiosome enriched with a Niemann-Pick type C2 (NPC2) transporter protein (Hambleton et al., 2019). The implication in sterol transport of NPC proteins are otherwise well characterized in mammalian lysosomal cholesterol metabolism (Strauss et al., 2002; Kidambi and Patel, 2008).

Dinoflagellates produce a specific 4-methylsterol called dinosterol and some other particular sterols like gorgosterol (C_30_ sterol products). These organisms use lanosterol as a sterol precursor (Shimizu et al., 1976; Alam, 1979; Volkman, 2016; Lu et al., 2020), and have evolved a so-called alternative squalene epoxidase to produce (3*S*)-2,3-oxidosqualene (Pollier et al., 2019). Dinosterol amounts are highly variable between dinoflagellates species in the genera *Symbiodinium*, *Gymnodinium*, *Fragilidium*, and *Gonyaulax* (Piretti et al., 1997; Amo et al., 2010). Dinosterol (4α,22,23-trimethylcholesta-5,22-dienol; Figure 3) has 3 exocyclic carbon atoms when compared to cholesterol. Sterols with an exocyclic methyl group at C4 are found in roundworms like *Caenorhabditis elegans* (Hannich et al., 2009; Zhou et al., 2020). In these invertebrates, which are sterol auxotrophs, dietary cholesterol is metabolized by several enzymes into 4α-methyllophenol just like in a cholesterol retro-biosynthetic segment. Among these enzymes, STRM-1 is a sterol-C4-methyltransferase (4-SMT) from the orthologous group of SMT (Zhou et al., 2020). 4-SMT has distinct specificity and substrate recognition characteristics when compared to SMT1 and SMT2 that catalyze methyl transfers on olefinic sterol side chains (Darnet et al., 2020). An orthologous 4-SMT sequence from *Symbiodinium* is reported in databases suggesting that 4-methylsterols in dinoflagellates originate from the same biosynthetic process functionally described in the case of round worms (Lu et al., 2020). The occurrence of 4-methylsterols produced through C4-methylation in very distant taxa suggest a convergent evolutionary process at play to gain biological functions, yet unclear in the case of dinoflagellates. *Fugacium kawagutii* exhibits a sterol profile with all compounds methylated at C4, and dinosterol and 24-desmethyldinosterol accounting for sixty percent of the total (Lu et al., 2020). Dinosterol has also two exocyclic carbon atoms in its side chain, at C24 and at the unusual C23 position for a sterol side chain alkylation, and a double bond at C22. This favors the existence of an additional SMT to SMT1, yet to be identified. Dinoflagellates may probably have a sterol-C22-desaturase reminiscent of the type of reaction catalyzed by a cytochrome P450 dependent monooxygenase in canonical pathways but this particular point requires further genome mining and functional assays in heterologous systems to uncover orthologous or novel side chain modifying enzymes. Besides dinosterol other sterol molecules like gorgosterol are considered as taxonomic markers of the dinoflagellates (Mackenzie et al., 1982). Gorgosterol is a very particular sterol not seen in canonical models but it features the wealth of complex sterol structures describes in marine organisms (Giner and Djerassi, 1991). Gorgosterol harbors a cyclopropyl group at C22 indicative of a side chain rearrangement most probably through reactions well studied in the case of cyclopropyl and cyclopropenyl sterols found in sponges (Giner, 1993). The sterolotype defined by dinoflagellates with a LAS and the particular 4-SMT in addition to SMT1 (Figure 2) would certainly urge further research effort to clarify a possible role of specific 4-methylsterols in the success of symbiotic association with the sterol auxotrophic cnidarians and this in the context of strong abiotic environmental stress leading to coral bleaching (Lu et al., 2020).

## Steroidogenic Enzymes as Targets for Antiparasitic Drugs

Two major groups found in Alveolata and Excavata (Burki et al., 2020) are the apicomplexan parasites, which are sterol auxotrophs, and the kinetoplastida *trypanosomatidae*, which are sterol autotrophs. The search for therapeutic chemicals has driven significant aspects of sterol research in these organisms particularly on sterol uptake and biosynthesis that are targets of unbroken medical and economic interest (Roberts et al., 2003; Chaudhary and Roos, 2005).

Species from the *trypanosomatidae* are causing severe parasitic diseases in human and other mammals like *T. cruzi* causes Chagas disease, *T. brucei* causes sleeping sickness (African trypanosomiasis), and Leishmania causes leishmaniasis. Trypanosomes synthesize their 24-alkylsterols *de novo* from isoprenic precursors produced through the mevalonate pathway (El-Sayed et al., 2005; Cosentino and Aguero, 2014; Gold et al., 2016). The conversion of lanosterol to ergosterol has been demonstrated in these parasites, which display therefore a fungal type of sterol pathway (Fügi et al., 2014; Gold et al., 2016). Some enzymes implied in this pathway especially those that are major targets for biocides have been the focus of intensive research. These enzymes are 3-hydroxy-3-methylglutaryl-CoA-reductase (HMGR), squalene epoxidase, lanosterol-C14-demethylase and sterol-C24-methyltransferase (SMT), inhibited by statins, allylamines, azoles, and azasterols, respectively, Buckner et al. (2000), Urbina et al. (2002), Roberts et al. (2003), Lepesheva et al. (2004, 2018), Friggeri et al. (2018), Darnet and Schaller (2019). Remarkably, a distinct sterol composition of specific parasitic developmental stages was described in *T. cruzi*. Epimastigotes contain ergosterol and amastigotes produce 24-methyl-Δ^7^-sterols and 24-ethylidene-Δ^7^-sterols (Cosentino and Aguero, 2014). In *T. brucei*, a complex network of ergosterol and novel ergostatrienols coined ETO [ergosta-5,7,25(27)-trien-3β-ol] and 24-DTO [dimethyl ergosta-5,7,25(27)-trienol] were detected in procyclic and bloodstream forms of the parasite (Nes et al., 2012). Amoeba and other metamonada are classified with the excavates (Burki et al., 2020). Some species present a sophisticated sterol metabolism that includes an endogenous sterol biosynthetic pathway and an additional capacity to metabolize exogenous cholesterol. *Naegleria lovaniensis*, *Naegleria gruberi*, and *Acanthamoeba polyphaga* are producing ergosterol, cholesterol, and poriferasterol derivatives (Raederstorff and Rohmer, 1987; Zhou et al., 2018). In these unicellulars, ergosterol biosynthesis occurs via cycloartenol (and not lanosterol), which is converted into 31-norlanosterol and from that point a pathway closely identical to the algae pathway is leading to the above-mentioned end-products. More recent studies combining chemistry and genomics in *Naegleria fowleri* confirmed a cycloartenol to cholesterol pathway in the amoeba, and also a dual origin of cholesterol of both biosynthetic and dietary origin (Zhou et al., 2018). *Naegleria* converts cholesterol into Δ^5,7^-dienic sterols most probably using a sterol-C7-desaturase of the Rieske oxygenase type, which has been initially discovered in sterol auxotrophs like *Drosophila* and round worms or *Tetrahymena*. These organisms require specific sterols at specific developmental stages of their life span and for this recruit specific enzymes yet considered as unusual with respect to the canonical view on sterol pathways.

## Diatoms and Other Stramenopila

The sterol content of diatoms has been reported (Volkman, 2003, 2016). The search for biomarkers of these planktonic organisms is motivated by the tremendous role they play in carbon dioxide fixation through photosynthesis and this at a global scale (Gold et al., 2017). An attempt to link a 18S RNA-based molecular phylogeny to sterol profiles of hundreds of species including raphid pennates, araphid pennates, radial centrics, and others came to the conclusive evidence that diatoms may not be specifically marked with a sterol profile (Rampen et al., 2010). In rare cases 23-methylsterols and 23,24-dimethylsterols were found, eventually distinguishing those species from other microalgae. The unusual sterol side chains with a methyl group at C-23, a 22-cyclopropane or a 24-propylidene is also found in dinoflagellates or sponges (Giner et al., 1991). In diatoms, the isoprenic precursors are provided by the plastidial MEP pathway but some species produce the C_5_ building blocks from mevalonate (Cvejic et al., 2000). The quite well studied *Phaeodactylum tricornutum* performs squalene epoxidation to (3*S*)-2,3-oxidosqualene using the alternative squalene epoxidase AltSQE that belongs to the fatty acid hydroxylase type of oxygenases, is widely distributed in many eukaryotic groups, and is distinct from the flavoprotein-type of squalene epoxidase (Pollier et al., 2019). Diatoms produce 24-alkyl-Δ^5^-sterols through the cycloartenol route (Fabris et al., 2014; Gallo et al., 2020). The effect of light spectral quality and temperature were shown as major physiological parameters acting on the sterol composition and quantity in *Phaeodactylum tricornutum* (Veron et al., 1996). *P. tricornutum*, *Thalassiosira* sp. and *Nannochloropsis oceanica* have been studied with an objective to assess their biotechnological potential for hydrocarbon or fine chemical industrial supply (Lu et al., 2014). Proteomic and chemical lipid profiling in these organisms unveiled particular aspects of sterol compartmentation and lipid homeostasis (Lupette et al., 2019). Interference with synthetic estrogens observed in a chemical screening for strong triacylglycerol producers in *P. tricornatum* resulted in a reallocation of acetyl-CoA building blocks from the isoprenoid/sterol pool to the fatty acyl-CoA pool (Conte et al., 2018). Two diatoms *Skeletonema marinoi* and *Cyclotella cryptica* were subjected to transcriptome profiling and sterol biosynthetic studies implementing ^13^C-glucose feeding to cultures (Gallo et al., 2020). Clionasterol (C24β-ethylcholesterol) and fucosterol were identified in these species (Gallo et al., 2020). A considerable amount of analytical work carried out on algae around the seventies has firmly established the distribution of epimeric pairs of C24-alkylsterols in eukaryotes as already depicted above, with pairs such as β-sitosterol (24α) and clionasterol (24β) found in higher plants and in algae, respectively. Likewise, the stereoisomers isofucosterol (*trans*C24) and fucosterol (*cis*C24) are found in higher plants and in algae, respectively, (Patterson, 1971). *In silico* analysis of the transcriptomes from *S. marinoi* and *C. cryptica* suggest that the formation of fucosterol is mediated by an algal SMT2 (in diatoms and red algae, therefore named red SMT), which may have diverged from an ancestor quite early in the evolution toward canonical SMT1 and SMT2 enzymes (Gallo et al., 2020). Alltogether the combination of AltSQE, CAS, and algal SMT2 defines an interesting diatom sterolotype (Figure 2). Strikingly, *P. tricornatum* is lacking a SMT2, which explains the absence of 24-ethylsterols in *P. tricornutum* (Rampen et al., 2010; Lu et al., 2014). The cycloartenol-to-cholesterol route was recently confirmed in the chaetocerid diatom *Chaetoceros muelleri* (Jaramillo-Madrid et al., 2020b).

Conjugation of sterols in diatoms is suggested to play a role in growth dynamics and particularly oceanic phytoplankton blooming, a process which is controlled by programmed cell death (Gallo et al., 2017). Sulfonation of sterols in diatoms may regulate blooming dynamics. In fact, organic extracts prepared from growth phase declining cultures of *S. marinoi* were used in bio-guided fractionation assays to isolate sterol sulfates. Cholesterol and dihydrobrassicasterol sulfates were found, but also sitosterol sulfate in the active fractions. In the latter case one may find intriguing that diatoms biosynthesize clionasterol (24β-ethylcholesterol) but use sitosterol and stigmasterol (24α-ethylsterols) as a substrate for sulfotransferases (Gallo et al., 2018; Nuzzo et al., 2018).

*Emiliania huxleyi* is a phytoplanktonic prymnesiophyte (haptophyte). This ubiquitous coccolithophore that is forming large blooms in the oceans has a tremendous impact on carbon and sulfur cycling, ecosystems, and ultimately on climate (Holligan et al., 1983; Rivero-Calle et al., 2015). *E. huxleyi* is certainly not the only unicellular phytoplanktonic alga with such an ecological importance (Rivero-Calle et al., 2015) but recent work showed that its growth and bloomings regulated by coccolithoviruses implied a considerable metabolic remodeling and reprogramming of the host lipid metabolism during the infection process (Rosenwasser et al., 2014; Frada et al., 2017). Major effects were seen on fatty acid and sphingolipid metabolism but also on the mevalonate/isoprenoid and sterol pathways apparently hijacked by the virus for its replication (Rosenwasser et al., 2014). The exact nature of all coccolithophore sterols was not reported in this study although previous chemotaxonomic studies established those sterol profiles in the context of geochemistry surveys (Taipale et al., 2016; Volkman, 2016). *E. huxleyi* produces a 24-ethylsterol (24-ethylcholesta-5,22E-dien-3β-ol) as a major component and small amount of cholesterol among other sterols (Volkman et al., 1981). Genome mining in this species points out an almost complete pathway and therefore the indication of a cycloartenol route in agreement with recent evolutionary studies (Gold et al., 2016).

## Sterol Pathways in Brown, Red, and Green Algae

Algae from diverse groups of phaeophyta (brow algae), rhodophyta (red algae), and chlorophyta (green algae) have triggered the interest of analytical biochemists since the initial characterization of fucosterol (*cis*-24-ethylidenecholesterol) in *Fucus vesiculosus* (Heilbron et al., 1934; Patterson, 1971; McKean and Nes, 1977; Heilbron and Kevles, 1988; Komura et al., 2014). Genome sequences have been produced for the brown algae *Ectocarpus siliculosus* (Cock et al., 2010) and *Saccharina japonica* (Ye et al., 2015). *E. siliculosus* exhibits a sterol profile with cholesterol, ergosterol and fucosterol as prominent compounds and also some 24-ethylsterols (Mikami et al., 2018). Sterol enzymes predicted from the *E. siliculosus* sequence were closely similar to those of the diatoms *Thalassiosira oceanica* and *T. pseudomona* (Lommer et al., 2012), however, the epoxidation of squalene to (3*S*)-2,3-oxidosqualene is done by the canonical squalene epoxidase, not by the alternative squalene epoxidase found in diatoms (Lu et al., 2014; Gold et al., 2016; Pollier et al., 2019). Two distinct SMTs were identified in genomes of *E. siliculosus* and *E. subulatus* sp. Ec32 genomes (Dittami et al., 2020), in agreement with sterol profiles. Further genome mining and experimental validation of candidate genes are required to document the apparent absence in these genomes of sterol-24-reductases (24RED) and sterol-22-desaturases (C22D) of the canonical type (Dittami et al., 2020). High amounts of fucosterol in brown algae are often accompanied by saringosterol, a 24-hydroxy-24-vinylcholesterol that arise from non-enzymatic oxidative conversion of fucosterol (Knights, 1970).

The red and brown algae synthesize cholesterol, with red algae being very strong producers (Tsuda et al., 1957; Gibbons, 1967). *Chondrus crispus* has more than 94% of cholesterol in its total sterols but desmosterol or Δ^22^-cholestenol were detected as well (Tasende, 2000). These species may represent a rich source of bio-based non-animal cholesterol. *C. crispus* and *Cyanidioschyzon merolae* were selected for in-depth genomic studies, from which a sterol pathway was inferred and modeled (Belcour et al., 2020). Sterol profiles were not established in the case of *C. merolae* but genomic datasets indicated the presence of a complete pathway from cycloartenol to cholesterol (Desmond and Gribaldo, 2009; Lu et al., 2014). The mixotrophic and thermophilic unicellular red algae *Galdieria sulphuria* and *Cyanidium caldarium* produce ergosterol and other dienic or polyenic ergosterol derivatives, in addition to 24-ethylsterols (Seckbach et al., 1993). Genomes of *G. sulphuria* and from *Porphyridium purpureum* have been sequenced, however, a curated catalog of sterol genes is not fully available, although annotations provided by databases (KEGG, MetaCyc, and others) indicate a cycloartenol route for sterol production in red algae and furthermore suggest a monophyletic evolutionary relationship between red algae and plants (Bhattacharya et al., 2013; Schonknecht et al., 2013).

Green algae from the chlorophyta like *Chlorella* sp., *Volvox* sp., *Chlamydomonas* sp., *Ostreococcus* sp., and some other genera like *Monodus* sp., and *Ochromonas* sp., from the chrysophyta (golden brown algae) have been studied decades ago (Moseley and Thompson, 1980; Gealt et al., 1981) with a continuous interest to depict the evolutionary relationship between, e.g., a fungal ergosterol pathway and an algal ergosterol pathway (Miller et al., 2012). The overall published data on algal sterols provides a clear-cut dichotomy of sterols structures found in large taxonomic groups (as already discussed in a previous section of this review): the sterol profiles established for all species of the chlorophyta and chrysophta exhibit C24β-methylsterols (brassicasterol, ergosterol) and C24β-ethylsterols (clionasterol, poriferasterol, and 7-dehydroporiferasterol). The technical requirements for the distinction of C24α - and C24β-ethylsterols (Rubinstein et al., 1976) most probably led to misnaming epimeric pairs of sterols here or there. Genome and proteomes of algae (Nguyen et al., 2011) are confirming the cycloartenol route for the production of 24-alkylsterols in green algae, which define a Chlorella-chlamydo-type of sterol pathways (Figure 2) (Miller et al., 2012). Pathways inferred from *in silico* analysis using canonical data sets as usual queries generally unclose orthologs and paralogs of steroidogenic enzymes. However, genes and enzymes acting on the sterol side chain modification (SMT, sterol-22-desaturases, sterol-24-reductase) or genes and enzymes required for the metabolization of the tetracyclic nucleus (sterone-3-reductase, sterol-C5-desaturase, sterol-C7-reductase and other putative enzymes) certainly deserve a further attention (Lu et al., 2014; Brumfield et al., 2017). Interestingly, the Chlamydomonas single SMT upon expression in yeast produces Δ^7,22^-poriferasterol, a C24β sterol epimer typical of algae (Brumfield et al., 2017). The access to novel sequence data for species like *Chlorokybus atmophyticus* (Wang et al., 2020) or *Penium maragaritaceum* (Jiao et al., 2020) will provide additional resources to enrich sterol profile records and to examine a sterol profile-genome congruence.

## Concluding Remarks

**FIGURE 7:**
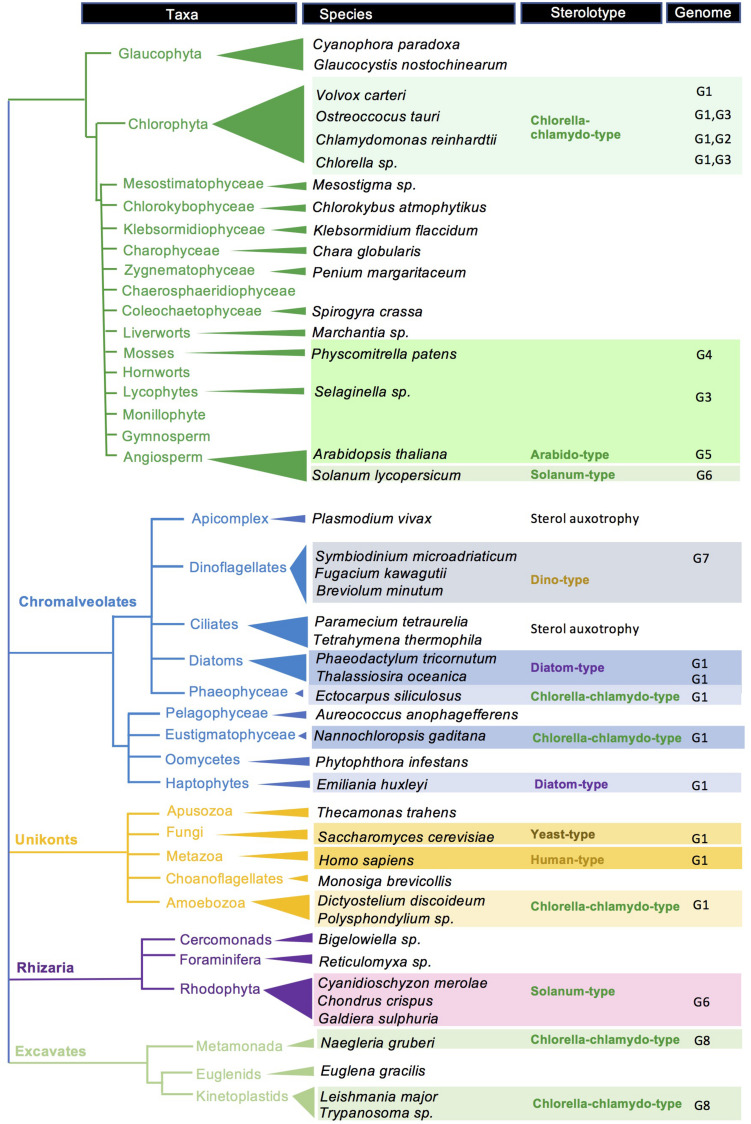
Sterolotype distribution over a tree of eukaryotes. A selection of species is tagged with sterolotypes presented in Figure 2. References about genomes and gene functions, and sterol profiles, are indicated by G1 to G8. G1, (Lu et al., 2014); G2, (Brumfield et al., 2017); G3, (Gold et al., 2016); G4, (Morikawa et al., 2009); G5, (Schaller, 2010); G6, (Sonawane et al., 2016); G7, (Lu et al., 2020); and G8, (Fügi et al., 2014). Species with no sterolotype indications are awaiting for a comprehensive mining of corresponding genomes. Gene nomenclature for *Arabidopsis thaliana*, *Saccharomyces cerevisiae*, *Homo sapiens*, and *Chlamydomonas reinhardtii* is given in Supplementary Table 1.

The access to novel sequenced genomes and the increasing quality of their expert annotation and curation is opening the search for interesting new genes, pathways and for a more exhaustive view upon the sterol biosynthetic capacities and distribution in eukaryotes (Figure 7). The integration of a considerable body of published data on the sterol composition of a multitude of species – sometimes in ancient and overlooked papers and repositories – with genome sequencing led us to tentatively visualize major taxonomic groups with sterolotypes, which may evolve as far as more information will be produced from genome surveys (Wong, 2019), especially those genomes coming from the oceans (Carradec et al., 2018; Canals et al., 2020). To reach this goal, indissociable efforts of investigating and validating accurate gene functions in dedicated heterologous expression systems will further support advanced phylogenomic studies (Desmond and Gribaldo, 2009; Gold et al., 2016). In the same vein, progresses in sterolomics will in turn provide users with powerful methods to envisage large-scale metaphenomics for a data-driven treatment of biological questions (Griffiths and Wang, 2019). Nonetheless, yet unknown genes and enzymes may well arise from forward and serendipitous genetic screenings. It is also worth noting that subtle variations in enzyme sequences like single amino acid substitution(s) may define a pathway, like for instance mutation(s) in cycloartenol synthase converts the latter into a LAS (Meyer et al., 2002). Consequently, genomic analyses *per se* are not sufficient to define sterolotypes, which are based on both decisive enzymes and accurate sterol compositions (Figure 2). The inherent limited potential of expert BLAST searches for predicting orthology and paralogy is especially emphasized when comparing protein sequences of selected species including canonical models, and enzyme-based sterolotypes. Species that belong to a same sterolotype as defined in this study display for each sterol biosynthetic enzymes a remarkably wide range of sequence identities (Supplementary Figure 1).

Sterolotypes defined in this paper readily stand on the AltSQE/SQE, CAS/LAS distinction, and on the sterol side chain modification enzymes (Figure 2). The exclusive choice of CAS or LAS is not fully understood as for instance the evolutionary conserved LAS in land plants has not been integrated as an essential contributor to sterol homeostasis. Sterolotypes flagged with CAS also highlight the remarkable conversion of cycloartenol to cholesterol in viridiplantae (Babiychuk et al., 2008; Ohyama et al., 2009; Sawai et al., 2014; Sonawane et al., 2016). This pathway recruits a particular sterol-Δ^24^-reductase paralog (SSR2 in *Solanum lycopersicum*) also found in rhodophyta (Calegario et al., 2016), for the synthesis of cholesterol. Distinct pathways for the production of that unique sterol in red algae or plants, and in mammals, speaks therefore in favor of convergent evolutionary process in sterol biology, as depicted in other eukaryotic groups in this brief overview.

Finally, sterols have raised interest from the biotech and industrial field due to their multiple pharmaceutical, cosmetic and food applications like blood cholesterol-lowering, anticancer, antiangiogenic, anti-inflammatory, antiatherosclerotic, anthelminthic, and antioxidant effects (Martin-Creuzburg and Elert, 2009; Martin-Creuzburg and Merkel, 2016). The sterol supply for a global market could possibly shift from agricultural waste products (Maniet et al., 2019) or plants (Zhang et al., 2020), to diatoms and other microalgae as a versatile system (Singh et al., 2017). Successful achievements in specific pathway engineering, for instance expected from the expression of limiting enzymes (Jaramillo-Madrid et al., 2020a), certainly require a further picturing of the intricate regulatory metabolic networks and cellular homeostasis of these producers (Marechal and Lupette, 2019), in order to provide at source to sink production of sterols compatible with the market demand (Luo et al., 2015).

## Author Contributions

SD and HS conceived the overview and wrote the manuscript with contributions from AB and QC. All authors contributed to the article and approved the submitted version.

## Conflict of Interest

The authors declare that the research was conducted in the absence of any commercial or financial relationships that could be construed as a potential conflict of interest.

## Supplementary Material

Supplementary Figure 1 Protein sequence comparison between species and sterolotypes. The orthologs were retrieved by BLAST search and the amino acid percent identity was reported in the matrix. Proteomes are imported from UniProt database. The accession numbers for *Homo sapiens*, HsLAS, *Saccharomyces cerevisiae*, ScLAS, ScSMT1, and *Arabidopsis thaliana*, AtCAS1, AtLAS1, AtSMT1, At plant SMT2 are indicated in Supplementary Table 1. Accessions for other protein sequences as follows. *Phaeodactylum tricornutum*: PtAltSQE, B7FXW1; PtCAS1, XP_002185678.1; PtSMT1, XP_002186194.1; Pt algal SMT2, XP_002178531.1; *Chlorella variabilis*: CvCAS1, XP_005848291.1; CvSMT1, XP_005845039.1; Cv algal SMT2, XP_005844298.1; *Symbiodinium microadriaticum*: SmCAS1, OLP86082.1; SmSMT1, OLP85101.1; Sm4-SMT, OLQ09145.1; SmAltSQE, CAE7673970.1; and *Solanum lycopersicum*: SlCAS1, NP_001233784.1; SlLAS1, XP_004240118.1; SlSMT1, XP_004229650.1; Sl plant SMT2, XP_004248642.1; SlSSR2, NP_001306251.1. Horizontal frames, species assigned to a sterolotype. Vertical frames, exemplified enzyme sequence diversity.
