# Supplementary figures and images for: Phytosterol Profiles, Genomes and Enzymes – An Overview
Source: Front Plant Sci. 2021 May 19;12:665206. doi: 10.3389/fpls.2021.665206 (PMC8172173; doi:10.3389/fpls.2021.665206)

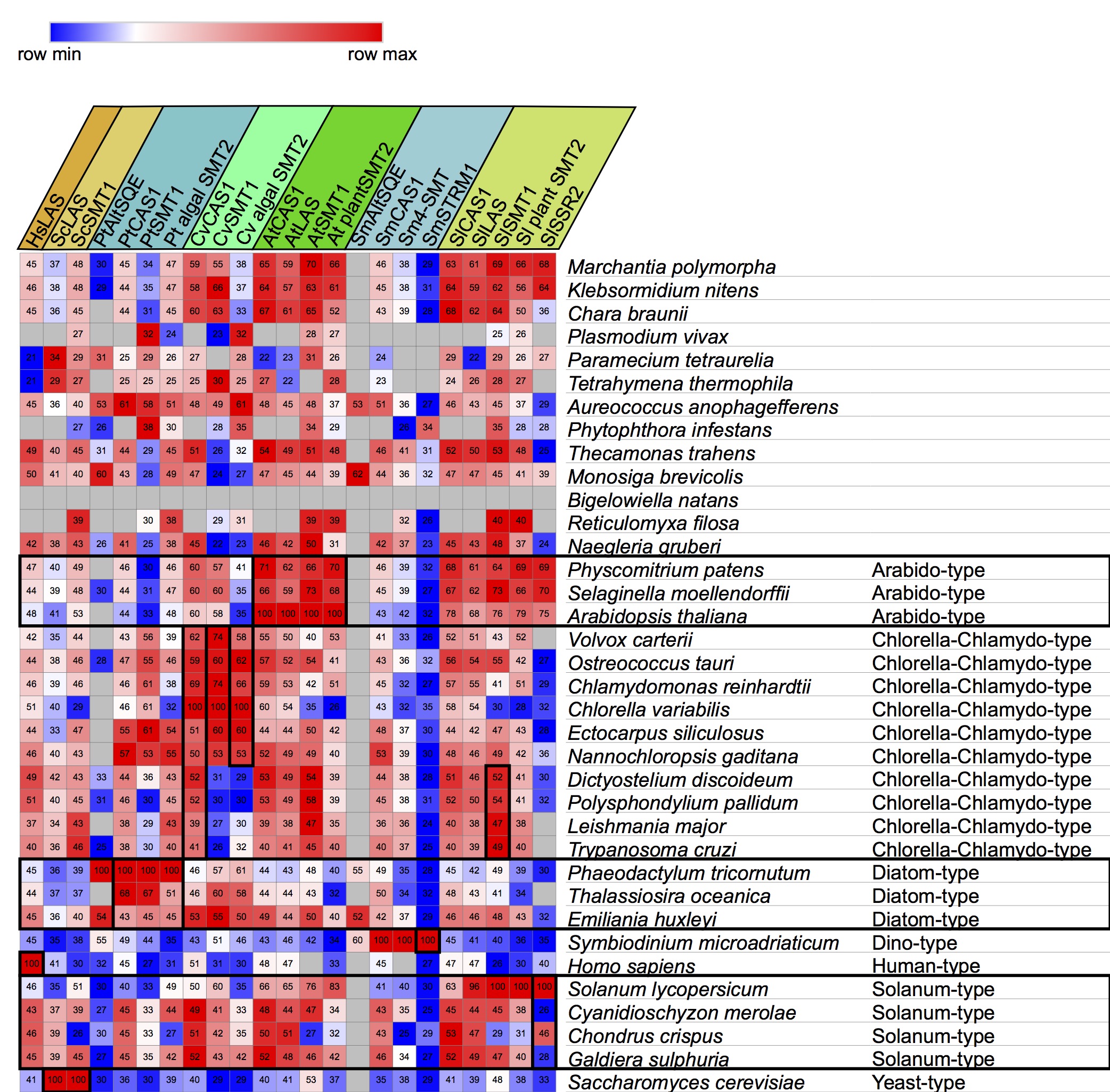

Supplement: Supplementary Figure 1 — Protein sequence comparison between species and sterolotypes. The orthologs were retrieved by BLAST search and the amino acid percent identity was reported in the matrix. Proteomes are imported from UniProt database. The accession numbers for Homo sapiens, HsLAS, Saccharomyces cerevisiae, ScLAS, ScSMT1, and Arabidopsis thaliana, AtCAS1, AtLAS1, AtSMT1, At plant SMT2 are indicated in Supplementary Table 1. Accessions for other protein sequences as follows. Phaeodactylum tricornutum: PtAltSQE, B7FXW1; PtCAS1, XP_002185678.1; PtSMT1, XP_002186194.1; Pt algal SMT2, XP_002178531.1; Chlorella variabilis: CvCAS1, XP_005848291.1; CvSMT1, XP_005845039.1; Cv algal SMT2, XP_005844298.1; Symbiodinium microadriaticum: SmCAS1, OLP86082.1; SmSMT1, OLP85101.1; Sm4-SMT, OLQ09145.1; SmAltSQE, CAE7673970.1; and Solanum lycopersicum: SlCAS1, NP_001233784.1; SlLAS1, XP_004240118.1; SlSMT1, XP_004229650.1; Sl plant SMT2, XP_004248642.1; SlSSR2, NP_001306251.1. Horizontal frames, species assigned to a sterolotype. Vertical frames, exemplified enzyme sequence diversity. [file Image_1.JPEG]
