# Supplementary material for: Phytosterol Profiles, Genomes and Enzymes – An Overview
Source: Front Plant Sci. 2021 May 19;12:665206. doi: 10.3389/fpls.2021.665206 (PMC8172173; doi:10.3389/fpls.2021.665206)
Supplement: Supplementary file 2 [file Table_1.DOCX]

| **Ab.** | **description** | **EC** | **A.thaliana** | **Yeast** | **Human** | **C.reinhardtii** |
| --- | --- | --- | --- | --- | --- | --- |
| SQS | squalene synthase | 2.5.1.21 | AT4G34640 (SQS1);  AT4G34650 (SQS2); | YHR190W (ERG9); | Chr 8: 11,795,573-11,839,309 fw (FDFT1); | Cre03.g175250.t1.2 |
| SQE | squalene monooxygenase / squalene epoxidase | 1.14.13.132 | AT1G58440 (SQE1,XF1);  AT2G22830 (SQE2);  AT4G37760 (SQE3);  AT5G24140 (SQE4,SQP2);  AT5G24150 (SQE5,SQP1);  AT5G24160 (SQE6); | YGR175C (ERG1); | Chr 8: 124,998,497-125,022,283 fw. (SQLE); | g17770.t1 |
| CAS | 2,3(S)-oxidosqualene-cycloartenol cyclase | 5.4.99.8 | AT2G07050 (CAS1); | --- | --- | Cre01.g011100 |
| LAS | lanosterol synthase | 5.4.99.7 | AT3G45130  (LAS1); | YHR072W (ERG7); | Chr 21: 46,188,141-46,228,824 rv. (LSS,OSC); | --- |
| SMT1 | C24 sterol methyltransferase | 2.1.1.41 | AT5G13710 (SMT1); | YML008C (ERG6); | --- | Cre12.g500500 |
| SMT2 | C28 sterol methyltransferase | 2.1.1.41 | AT1G20330 (SMT2-1,SMT2);  AT1G76090 (SMT2-2,SMT3); | --- | --- | --- |
| SMO | C-4 methyl sterol oxidase | 1.14.13.72 | --- | YGR060W (ERG25); | Chr 4: 165,327,623-165,343,160 fw. (MSMSO1,DESP4, SC4MOL, ERG25); | Cre06.g261200.t1.2 |
| SMO1 | 24-methylenecycloartanol 4α-methyl oxidase | 1.14.13.72 | AT4G12110 (SMO1-1);  AT4g22753 (SMO1-2);  AT4g22756 (SMO1-3); | --- | --- | --- |
| SMO2 | 4α-methyl-Δ7-sterol-4α-methyl oxidase | 1.14.13.72 | AT1G07420 (SMO2-1);  AT2G29390 (SMO2-2); | --- | --- | --- |
| βHSD | 3β-hydroxysteroid dehydrogenase/C4-decarboxylase | 1.1.1.170 | AT2G33630 (AT2G33630.1);  AT2G43420 ();  AT1G47290 (AT3BETAHSD/D1,CSD);  AT2G26260 (AT3BETAHSD/D2); | YGL001C (ERG26); | Chr X: 152,830,967-152,869,729 fw (NSDHL,XAP104, SDR31E1, H105e3); | Cre10.g442200.t1.2 |
| SR | sterone ketoreductase | 1.1.1.100 | At5g18210  (SKR); | YLR100W (ERG27); | Chr 1: 162,790,702-162,812,817 fw. (HSD17B7,SDR37C1, PRAP); | ? |
| CPI | cyclopropyl sterol isomerase | 5.5.1.9 | AT5G50375 (CPI1); | -- | -- | Cre16.g657300.t1.2 |
| C14DM | obtusifoliol-14α-demethylase | 1.14.13.70 | AT1G11680 (OBT14DM1,CYP51G1);  AT2G17330 (OBT14DM1,CYP51G2); | YHR007C (ERG11); | Chr 7: 92,112,151-92,142,952 rv. (CYP51A1); | Cre02.g092350.t1.2 |
| 7ISO | Δ8-Δ7-sterol isomerase | 5.3.3.5 | AT1G20050 (HYD1); | YMR202W (ERG2); | Chr X: 48,521,158-48,528,716 fw. (EBP,CPXD, CPX, CHO2, CDPX2); | Cre12.g557900.t1.2 |
| C14R | Δ8,14-sterol-Δ14-reductase | 1.3.1.70 | AT3G52940 (FK); | YNL280C (ERG24); | Chr 1: 225,401,502-225,428,925 rv. (DHCR14,LBR); | Cre02.g076800.t1.2 |
| C5D | Δ7-sterol-C5-desaturase | 1.14.19.20 | AT3G02580 (C5DES1,STE1,DWF7,BUL1);  AT3G02590 (C5DES2,STE2); | YLR056W (ERG3); | Chr 11: 121,292,453-121,308,694 fw. (SC5D,SC5DL); | Cre16.g663950.t1.2 |
| C7R | Δ5,7-sterol-Δ7-reductase | 1.3.1.21 | AT1G50430 (DWF5); | --- | Chr 11: 71,428,193-71,452,868 rv. (DHCR7,SLOS); | --- |
| C24RI | Δ5-sterol-Δ24-reductase (isomerase) | 1.3.1.72 | AT3G19820 (DWF1); | --- | --- | --- |
| C24R | Δ5-sterol-Δ24-reductase |  | --- | YGL012W (ERG4); | Chr 1: 54,849,633-54,887,218 rv. (DHCR24,KIAA0018, DCE, seladin-1); |  |
| C25R | sterol-Δ25-reductase |  | --- | --- | --- | ? |
| C22D | C-22 sterol desaturase | 1.14.19.41 | AT2G28860 (AT2G28860,CYP710A4);  AT2G28850 (AT2G28850,CYP710A3);  AT2G34500 (CYP710A1);  AT2G34490 (CYP710A2); | YMR015C (ERG5); | --- | g11457.t1 |
| ERG28 | complex anchor | - | AT1G10030 (ERG28); | YER044C (ERG28); | Chr 14: 75,649,791-75,661,189 rv. (ERG28,C14orf1, NET51); | Cre13.g567900.t1.2 |
| USGT | UDP-glucose: sterol glucosyltransferase | 2.4.1.173 | AT3G07020 (UDP1,SGT);  AT1G43620 (UDP2,UGT80B1); | --- | --- | g15030.t1; g9716.t1;   Cre07.g333450.t1.2;   Cre03.g207550.t1.2;   Cre03.g207800.t1.2; |
| ASAT1 | acyl-CoA sterol acyl transferase 1 | 2.3.1.- | AT3G51970 (SAT1); | YCR048W (ARE);  YNR019W (); | Chr 1: 179,293,714-179,358,680 fw. (SOAT1,ACAT, SOAT, STAT);  Chr 12: 53,103,518-53,124,538 fw. (SOAT2,ACAT2); | Cre02.g098050.t1.2;  Cre07.g349900.t1.3;   Cre05.g240050.t1.3;  Cre16.g685300.t1.2;  Cre07.g350050.t1.3;  Cre07.g349650.t1.2;  Cre06.g308700.t1.2;  Cre12.g548600.t1.3; |
| PSAT | Phospholipid--sterol O-acyltransferase | 2.6.1.52 | At1g04010 (LCAT2,PSAT1); | --- | --- | ? |
